# Involvement of LDL and ox-LDL in Cancer Development and Its Therapeutical Potential

**DOI:** 10.3389/fonc.2022.803473

**Published:** 2022-02-16

**Authors:** Chang-Feng Deng, Neng Zhu, Tan-Jun Zhao, Hong-Fang Li, Jia Gu, Duan-Fang Liao, Li Qin

**Affiliations:** ^1^Division of Stem Cell Regulation and Application, School of Pharmacy, Hunan University of Chinese Medicine, Changsha, China; ^2^Department of Urology, The First Hospital of Hunan University of Chinese Medicine, Changsha, China; ^3^Institutional Key Laboratory of Vascular Biology and Translational Medicine in Hunan Province, Hunan University of Chinese Medicine, Changsha, China

**Keywords:** tumorigenesis, cancer development, LDL, ox-LDL, statins

## Abstract

Lipid metabolism disorder is related to an increased risk of tumorigenesis and is involved in the rapid growth of cancer cells as well as the formation of metastatic lesions. Epidemiological studies have demonstrated that low-density lipoprotein (LDL) and oxidized low-density lipoprotein (ox-LDL) are closely associated with breast cancer, colorectal cancer, pancreatic cancer, and other malignancies, suggesting that LDL and ox-LDL play important roles during the occurrence and development of cancers. LDL can deliver cholesterol into cancer cells after binding to LDL receptor (LDLR). Activation of PI3K/Akt/mTOR signaling pathway induces transcription of the sterol regulatory element-binding proteins (SREBPs), which subsequently promotes cholesterol uptake and synthesis to meet the demand of cancer cells. Ox-LDL binds to the lectin-like oxidized low-density lipoprotein receptor-1 (LOX-1) and cluster of differentiation 36 (CD36) to induce mutations, resulting in inflammation, cell proliferation, and metastasis of cancer. Classic lipid-lowering drugs, statins, have been shown to reduce LDL levels in certain types of cancer. As LDL and ox-LDL play complicated roles in cancers, the potential therapeutic effect of targeting lipid metabolism in cancer therapy warrants more investigation.

## Introduction

Cholesterol is an indispensable component of life, and the intracellular cholesterol levels are maintained through a series of factors, including cholesterol synthesis, uptake, efflux, esterification, metabolism, and transportation (1). Epidemiological studies have shown that cholesterol plays a vital role in the occurrence and development of cancer, and high plasma cholesterol levels are positively correlated with the death risk of certain cancer types (2). It has been reported that every 10 mg/dL increase in cholesterol increases the risk of recurrence of prostate cancer by 9% (3). With the rapid proliferation of tumors, cancer cells need large amount of cholesterol to meet membrane biogenesis and biofunctional requirements (4). Statins mainly exert lipid-lowering effect through mevalonate pathway. Studies have shown that inhibition of mevalonate pathway can down-regulate the expression of farnesyl pyrophosphate (FPP) and geranylgeranyl pyrophosphate (GGPP) isoprenyls, the isoprenyl group is critical in modifying G proteins involved in cancer cell proliferation, migration, and survival (5). Various studies support the positive roles of statins in human cancer suppression or patient’s prognosis (6–8), and the statin users’ cancer-related mortality and recurrence rate are significantly reduced (8).

**Figure 1:**
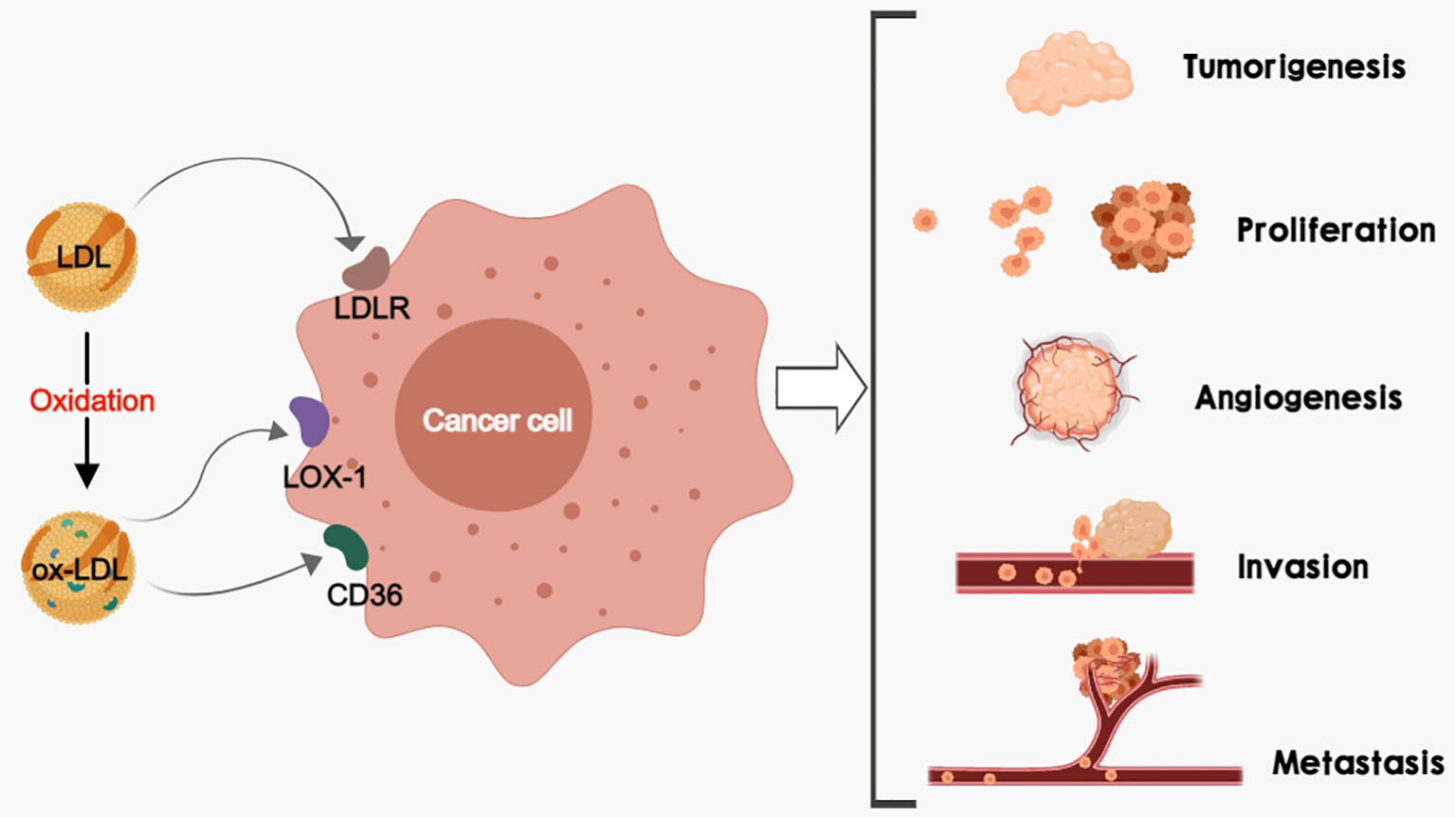
LDL and/or ox-LDL bind to their receptors (LDLR, LOX-1, and CD36) respectively to promote tumorigenesis, cancer cell proliferation, cancer angiogenesis, as well as cancer invasion and metastasis.

In the process of tumorigenesis and progression, cancer cells exhibit metabolic abnormalities to meet the elevated energy and biosynthetic demands associated with the rapid growth of tumors (9). It is worth mentioning that cholesterol is the precursor for steroid hormones, bile acids, vitamin D, and oxysterols, and acts as a key material for cell growth (10). Low-density lipoprotein (LDL) is a critical lipoprotein and carrier of cholesterol mediating the transfer of cholesterol from the liver to peripheral tissues (11). When cellular cholesterol levels decrease, the expression of LDL receptor (LDLR) increases, and the extracellular domain of LDLR can bind to circulating LDL and promote its uptake through endocytosis. After entering the cell, LDL will be delivered to the lysosome. LDL is hydrolyzed by lipases, and then free cholesterol is released for cell utilization (12). Recently, LDLR has been found to be over-expressed in various cancers such as hepatocellular carcinoma (HCC), lung cancer, breast cancer, colorectal cancer, prostate cancer, and so on (13–15). Since cancer cells require more cholesterol to obtain energy than normal cells, they may raise their cholesterol levels through receptor-mediated endocytosis of LDL (16). Abnormal lipid metabolism can produce lipotoxicity that induces oxidative stress, which can significantly increase reactive oxygen species (ROS) levels (17), Gradual increase in oxidative stress can lead to the oxidation of intracellular LDL to oxidized low-density lipoproteins (ox-LDL). Besides, oxidative stress promotes DNA damages in cancers, which further results in malignant transformation and carcinogenesis (18, 19). Elevated plasma ox-LDL has been detected in breast cancer, gastric cancer, and colon cancer (20, 21). Since different types of cancer present different lipid disorders, the lipid may play versatile roles according to the types of cancer (22). Therefore, LDL and ox-LDL may have variable effects during cancer development (**Figure 1**).

### LDL and ox-LDL in Lipid Homeostasis

LDL is a complex particle containing proteins and lipids, and its outermost layer is surrounded by a lipid core and monomeric protein ApoB-100 (23). Cholesterol synthesized by the liver is transported to cells throughout the body by LDL, and about 70% of LDL-cholesterol (LDL-C) in plasma is degraded by LDLR-mediated endocytosis (24). LDLR activity is the primary factor determining circulating LDL levels. Due to its critical role in cholesterol homeostasis, LDLR mediates various signaling transduction in hepatocytes. Moreover, sterol regulatory element-binding proteins (SREBPs) are transcription factors for cholesterol production and absorption, and they regulate one of the critical transcription pathways involved in cholesterol homeostasis (25, 26). LDLR and several enzymes related to cholesterol synthesis, including recombinant 3-hydroxy-3-methylglutaryl coenzyme A reductase (HMGCR) and squalene epoxidase (SQLE), are directly regulated by SREBP (9). Once LDL is endocytosed, it fuses with lysosomes and then is hydrolyzed in the lysosome to release cholesterol, fatty acids, and amino acids. When cellular cholesterol levels are low, SREBP2 is cleaved and transferred to the nucleus, where it up-regulates the expression of LDLR; when cellular cholesterol levels are high, SREBP2 remains inactive, and LDLR expression is down-regulated (27, 28). Proprotein convertase subtilisin/kexin type 9 (PCSK9) is another key regulator of LDLR. PCSK9, as a liver protease, can promote the degradation of hepatic LDL receptors, leading to increased LDL-C levels (29–31). Up to date, PCSK9 inhibitors evolocumab and alirocumab have been successfully applied to reduce circulating LDL-C levels (32). Excessive intracellular cholesterol can be esterified by acyl-coenzyme A (cholesterol acyltransferases) and stored in lipid droplets. Meanwhile, it can also be transported to the blood through ATP binding cassette transporters A1 (ABCA1) or ATP binding cassette transporters G1 (ABCG1), both ABCA1 and ABCG1 are transcriptionally regulated by the Liver X Receptor (LXR) (33). LXR helps maintain cholesterol homeostasis not only through promotion of cholesterol efflux but also through suppression of LDL uptake by enhancing E3 ubiquitin ligase activity and mediating LDLR degradation (34).

LDL contains polyunsaturated fatty acids, which can be oxidized by ROS and reactive nitrogen species (RNS) to generate lipid peroxides, such as ox-LDL. Whereas ox-LDL, in turn, stimulates ROS production (35). ApoB-100 is the protein component of LDL, as well as a high-affinity ligand for LDLR (36). Cysteine, lysine, histidine, and tyrosine residues in ApoB-100 are also the oxidation targets of ROS and RNS, and the oxidative modification of ApoB-100 may abrogate its function as an LDLR ligand (37). Once ox-LDL is no longer recognized by LDLR, it may be identified and combined with scavenger receptors (SRs) such as lectin-like oxidized low-density lipoprotein receptor-1 (LOX-1), scavenger receptor A (SR-A), and cluster of differentiation 36 (CD36). Ox-LDL is a well-known biomarker for cardiovascular diseases, and it enhances endothelial cell adhesion by activating oxidative stress and stimulates the expression of pro-inflammatory factors and adhesion molecules, as well as chemokines in vascular endothelial cells, leading to endothelial dysfunction (38). In recent years, more and more studies have focused on ox-LDL and cancers, and it has been found that the elevated levels of ox-LDL, as well as LOX-1 and CD36, are related to the increased risk of various cancers. Ox-LDL promotes epithelial-mesenchymal growth, cytoplasmic transformation, induces protective autophagy, activates inflammasomes, and the promotes release of growth factors, cytokines, and other pro-inflammatory markers to stimulate oncogenic signals, resulting in cell mutations and chemotherapy resistance (39).

### LDL and ox-LDL in Cancer Development

Alterations in blood cholesterol levels (decreased or increased) are critical phenomena in many malignancies (2). Hyperlipidemia has been shown to increase the risk of cancer (40), and cancer cells tend to accumulate a high amount of cholesterol either by up-regulating cholesterol biosynthesis or by enhancing cholesterol uptake for rapid cancer development (41). Increased intracellular cholesterol content has been observed in the tissues of breast cancer, ovarian cancer, and renal cancer (42, 43). Overproduction of LDLR is an important mechanism for cancer cells to obtain more essential fatty acids through LDLR endocytosis. It has been found that the up-regulation of LDLR can promote the rapid uptake of LDL in most cancers. The expression of LDLR is affected by feedback regulation of LDL-C levels in normal human prostate cells, while this feedback regulation is commonly lost in prostatic cancer cells (44).

**Figure 2:**
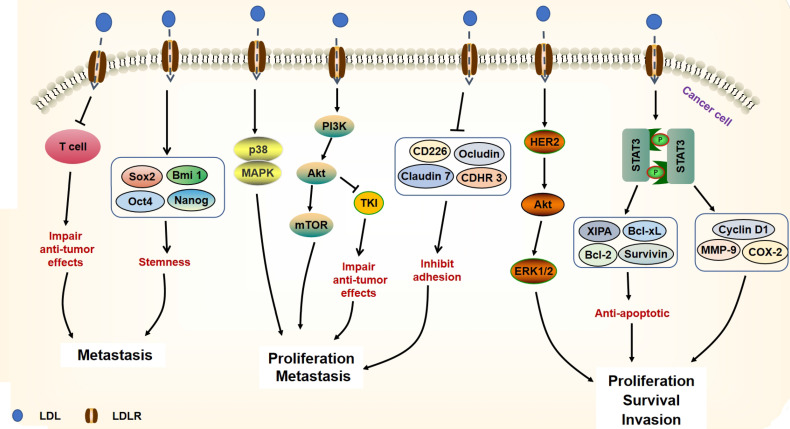
Signaling pathways of LDL on cancer progression. The accumulation of LDL can damage the anti-tumor effects of T cells. Through up-regulating the expressions of stemness genes (Sox2, Bmi 1, Oct4, Nanog), LDL supports cancer metastasis. LDL activates p38 and MAPK as well as PI3K/Akt/mTOR signaling pathways, leading to cancer proliferation and metastasis. Besides, through activation of PI3K/Akt signaling, LDL compromises the TKI anti-tumor efficacy against cancer cells. LDL decreases adhesion molecules (CD226, Ocludin, Claudin 7, CDHR3), which help cell migration. Moreover, LDL can activate HER2/Akt/ERK signaling pathway and up-regulate STAT3 target genes (including anti-apoptotic genes and MMP-9, Cyclin D1, COX-2) expression, which promote the survival and invasion of cancer cells.

Epithelial mesenchymal transformation (EMT) is a process of losing epithelial apical-basal polarity and cell-cell adhesion and transiting to invasive mesenchymal cells. After EMT, cells possess a number of malignant properties to carcinoma cells, including invasive behavior, stemness, and greater resistance to chemotherapy and immunotherapy (45). Many EMT transcription factors are regulated by PI3K/Akt and ERK signaling pathways to promote cancer cell proliferation and migration (46). In addition, PI3K/Akt regulates cancer cell growth by activating mTOR, which may promote cholesterol synthesis and uptake by activating SERBP. STAT3 activation is associated with transcription of genes involved in cell proliferation, migration, and survival, as well as increased expression of vascular endothelial growth factor (VEGF) and matrix metalloproteinases (MMPs) that favor angiogenesis (47). Preclinical studies have highlighted the importance of LDL in supporting the growth and proliferation of different cancer types by tuning numerous signaling pathways (PI3K/Akt, ERK, STAT3, etc.) (48–50). Alternatively, with high LDL levels, tumors may evade immune surveillance; LDL has been shown to limit the antitumor therapeutic effect of human γδ T cells *in vivo*, thereby enhancing tumor metastasis (51). In line with the results, chronic lymphocytic leukemia patients show a high incidence of elevated LDL cholesterol and their survival rates have improved after treatment with statins (52). Clinical data have shown that higher levels of cholesterol and LDL are associated with lower overall survival (OS) of patients treated with anti-PD1/L1 (53). Moreover, LDL cholesterol promotes the lymph node metastasis of colon cancer cells by inducing the activation of microvascular endothelial cells (54). It is also worth noting that LDL has been found to enhance cell stemness (55). Overall, these studies reveal that LDL has deleterious effects on cancer development (**Figure 2**).

Elevated levels of ox-LDL are a significant feature of lipid metabolism disorders and inflammation and have been implicated in many aspects of cancer, ox-LDL has been shown to induce mutagenesis, stimulate proliferation, induce autophagy, and initiate metastasis. For example, Esterbauer et al. showed that components of ox-LDL such as 4-hydroxynonenal could stimulate primary rat hepatocytes mutagenesis *in vitro* (56). In addition, ox-LDL can potentially contribute to the induction of cancer by increasing the expression of microRNA-210 (miR-210) (57). Ox-LDL upregulates hypoxia-inducible factor-α (HIF-α) expression and increases miR-210 expression, which leads to downregulation of sprout-related EVH1 domain 2 (SPRED2), a protein that reduces cell migration, leading to a higher risk of cancer and vascular diseases (58). Other studies have shown that administration of ox-LDL increases the proliferation of patient-derived glioblastoma xenografts and ovarian carcinoma cells (59, 60). These data support an effect of ox-LDL on promoting tumor growth.

Autophagy is an evolutionarily conserved intracellular self-defense mechanism, and organelles and proteins are degraded into autophagy bubbles through fusion with lysosomes. Cells thereby prevent the toxic accumulation of damaged or unnecessary components, but also recycle these components to sustain metabolic homoeostasis (61). Recent studies suggest that autophagy is a powerful survival strategy for cancer cells, by recycling intracellular components in conditions of metabolic stress or during anticancer treatments (62). Autophagy is an important mechanism for ox-LDL to participate in cancer progression. Ox-LDL activates the key metabolic enzyme oproline oxidase (POX) and promotes cancer cell autophagy through the mechanism related to ox-LDL and PPARγ. This study also found that the effect of POX on autophagy was achieved by producing superoxide that took effects by regulating beclin-1 (63). Another study showed that ox-LDL was capable of inducing autophagy in part through activation of microRNA155 (miR-155) in HUVEC cells (64). By activating autophagy, cancer cells undergoing EMT can gain resistance to cell death as a strategy for survival when spreading outside the tumor mass (65).

**Figure 3:**
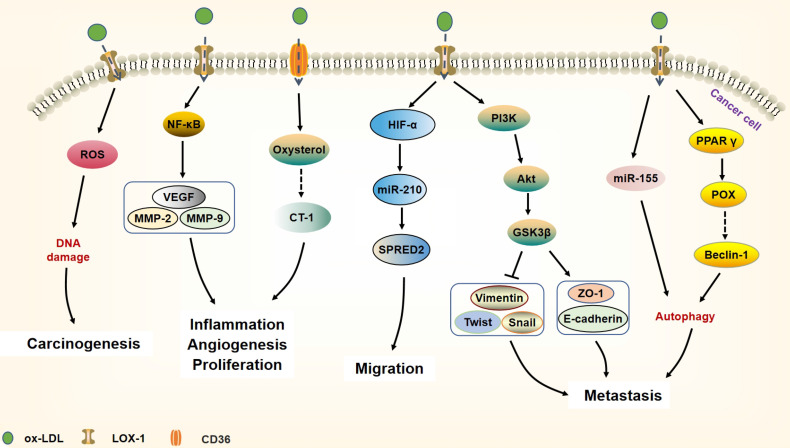
Ox-LDL binds to its receptor CD36 or LOX-1 and participates in tumorigenesis and development. Ox-LDL can cause DNA damage by generating ROS, thereby increasing the risk of carcinogenesis. Besides, ox-LDL binds to LOX-1 and activates NF-κB target genes (VEGF, MMP-2, MMP-9) to increase cell proliferation, motility, and angiogenesis. In addition, ox-LDL is also internalized by CD36 and further accumulates oxysterol metabolites, leading to the cytokine CT-1 expression that can promote inflammation and angiogenesis in cancer. Ox-LDL increases the expression of miR-210 by activating the expression of HIF-α and then down-regulating SPRED2, which leads to cancer cell migration. Furthermore, through triggering the PI3K/Akt/GSK3β cascade, ox-LDL can promote EMT in cancer. Autophagy is an important mechanism of ox-LDL involved in cancer EMT, ox-LDL induces cancer cell protective autophagy by activating miR-155 and regulating prolinase POX. Through the activation of POX, ox-LDL produces superoxide and up-regulates beclin-1, thereby mediating cancer cell autophagy.

Recent evidence suggests that as specific receptors for ox-LDL, CD36, LOX-1 are upregulated in and contribute to the pathophysiology of dyslipidemia-related diseases, such as cardiovascular disease and obesity (66, 67). Studies have found that LOX-1 is upregulated and promotes tumor development in different cancers such as breast cancer, colorectal cancer, and ovarian cancer. Together, the combination of ox-LDL and LOX-1 stimulates ROS production, which leads to oxidative DNA damage (39). Besides, it can promote cancer cell proliferation, invasion, and angiogenesis by activating nuclear factor kappa-B (NF-κB) and up-regulating the expression of *VEGF, MMP-2*, and *MMP-9* (20, 39). The scavenging receptor CD36, a scavenger receptor for ox-LDL, is found to be highly expressed in multiple cell types and mediates lipid uptake, immunological recognition, inflammation, molecular adhesion, and apoptosis (68). Furthermore, addition of ox-LDL has been shown to stimulate the proliferation of glioblastoma patient-derived xenografts, whereas siRNA-mediated knockdown of CD36 resulted in reduced proliferation. Ox-LDL is internalized by CD36 and the accumulation of ox-LDL and oxysterol metabolites can lead to overexpression of carditorphin 1(CT-1), which subsequently promotes inflammation, proliferation, and angiogenesis (60). Park et al. showed that the binding of ox-LDL to CD36 enhanced the activation of focal adhesion kinase 1 (FAK1) and Ras-related C3 botulinum toxin substrate 1 (RAC1) (69), which might be in part responsible for the cellular morphological changes necessary for the initiation of EMT, including loss of polarity and actin polymerization (70). As an important signal membrane transporter, CD36 is involved in the uptake of ox-LDL and binds with ox-LDL to participate in EMT signal transduction. All of these effects are shown in **Figure 3**.

## Dissecting the Roles of LDL and ox-LDL in Particular Types of Cancers

### LDL and ox-LDL in Breast Cancer

Breast cancer is the second leading cause of cancer-related deaths in females (71). Breast cancer is a heterogeneous disease, with major subtypes defined by expression of estrogen receptor (ER), progesterone receptor (PR), and epidermal growth factor receptor 2 (HER2) receptor (72). The major subtypes are as follows: luminal (ER-positive), HER2-like (mainly ER-negative and HER2-positive), and basal-like (mainly ER-negative, PR-negative, and triple-negative). The HER2-like and basal-like are the most aggressive and these subtypes are often used to predict prognosis and treatment responses (73).

A positive correlation between LDL-C and breast cancer progression has been observed. A prospective study in Portugal showed that plasma LDL-C levels were positively correlated with the tumor volume, breast cancer patients with higher levels of LDL-C at diagnosis have larger tumors, higher differentiation grade, and proliferative rate (74). Two Mendelian randomized studies support the finding that the increased plasma LDL-C is associated with a higher risk for breast cancer (75, 76). In contrast, two studies found that LDL-C was negatively associated with breast cancer risk (77, 78). Confounding factors such as region, diet, and comorbidities, as well as individual researchers’ biases in the selection of populations and measurement methods, might have contributed the differences in clinical studies (79).

The LDL-C exerts its effects on breast cancer cells through a variety of mechanisms. LDL promotes the proliferation and migration of ER-negative cell lines, but not ER-positive cancer cells. LDL-C can also induce the proliferation of the HER2-positive breast cancer cell line BT-474 (48). Clinicopathological studies have shown that with the increase in LDLR and acyl coenzyme a-cholesterol acyltransferase-1 (ACAT-1), the accumulation of cholesterol ester increases accompanied by the advancement of tumor grade (80). The increased LDL-C internalization and esterification may explain these differences in HER2-like or triple-negative breast cancer cells. They present with increased expression of LDLR and increased expression and activity of ACAT-1, which lead to a more remarkable ability to absorb, store, and utilize exogenous cholesterol in cells (81). A potential mechanism for LDL-promoted breast tumorigenesis is to increase phosphorylation of oncogenic signaling pathways, Akt and ERK *via* activating HER2 (48, 82). LDL also promotes the progression and metastasis of breast cancer through Akt-induced EMT and angiogenesis, increases the levels of mesenchymal markers *Slug, vimentin*, and *β-catenin*, and decreases in expression of adhesion molecules (*cadherin-related family member3(CDHR)*, *CD226*, *Claudin 7* and *Ocludin*), thereby promoting the migration and invasion of breast cancer cells (48, 83). Beyond *in vitro* studies, Gallagher et al. found that elevated LDLR expression in tumor accelerated LDL-C-mediated breast cancer growth in hyperlipidemic mice, In contrast, LDLR silencing and lower circulating levels of LDL-C retard tumor growth in HER2 positive and triple-negative breast cancer mouse models (84). So far, ample studies have emphasized the importance of LDL in breast cancer occurrence and development.

It has been found that elevated levels of ox-LDL are detected in the plasma of breast cancer patients, and the elevated plasma ox-LDL levels are positively correlated with the increase of breast cancer risk (85, 86). In addition, as the main receptor for internalization of ox-LDL, LOX-1 is overexpressed in 70% of human breast cancers and has been shown to be positively correlated with tumor grade and stage (87). Ox-LDL promotes the proliferation of the non-tumorigenic mammary epithelial cell line MCF10A cells and up-regulates the pro-inflammatory signals. Concretely, ox-LDL stimulates the proliferation of breast cancer cells *via* miR-21 in a dose-dependent manner, thereby activating the PI3K/Akt signaling pathways (88). Additionally, hominoid-specific oncogene, TBC1D3, a protein that regulates migration of human breast cancer cells have been found to up-regulate LOX-1 expression by activating the TNFα/NF-κB signaling. Ox-LDL binds to LOX-1 and activates inflammatory pathways through NF-κB, leading to transformation (13, 88). In contrast, depletion of LOX-1 by siRNA inhibits the invasion and migration of transformed breast mammary epithelial cells (13). Similarly, inhibition of LOX-1 by an antibody or a recombinant LOX-1 protein substantially suppresses the transendothelial migration of human breast cancer cells (89–91). Furthermore, LOX-1Δ4 is a splice variant of LOX-1 expressed in humans that lacks exon 4 (92). Due to the specific metabolic environment of different breast cancer phenotypes, LOX-1 and its splicing variant LOX-1Δ4 may play a carcinogenic role in the specific regulation of expression patterns. A full understanding of LOX-1 and LOX-1Δ4 molecular pathways in breast cancer may help develop a possible therapeutic option specific for different phenotypic cancer subtypes (87). As indicated above, a more in-depth study of ox-LDL and LOX-1 as potential mediators for the cholesterol-breast cancer link should be performed.

### LDL and ox-LDL in Colorectal Cancer

Recently, the field of cancer research has directed increased interest towards subsets of obesity-associated cancers. Specifically, data shows that in countries with high obesity prevalence, colorectal cancer incidence is high (93). Body mass index (BMI) is an important indicator of the survival of cancer patients, including colorectal cancer (94).

High levels of LDL-C are associated with increased colorectal cancer risk (95, 96), whereas the levels of free cholesterol and LDL-C in serum are found to be significantly lower in patients with lymph node metastasis than in patients without lymph node metastasis (97). Similarly, LDL-C acts as an independent prognostic factor for poor prognosis in metastatic colorectal cancer patients (98), and these results corroborate that higher LDL-C promotes distant metastasis in patients with colorectal cancer (98, 99). LDLR is essential for transporting serum LDL into cells. Several studies have evaluated the LDLR expression in colorectal cancer and have observed increased expression in colorectal cancer tissues, especially in colorectal cancer patients at stage N and M (55, 100). Serum LDL-C levels in advanced cancer patients decreased due to the increased metabolic demands in cancer cells. Therefore, a decreased in blood cholesterol level in colorectal cancer patients may be a consequence of increased uptake of blood cholesterol by cancer cells; it is less likely to be a cause for colorectal cancer initiation (101). Study also showed that LDL could enhance stemness by increasing stemness-related genes, such as *Sox2, Oct4, Nanog*, and *Bmi1* in colorectal cancer cells, increased ROS levels that can further activate MAPK pathways, and stimulated intestinal inflammation and colorectal cancer (55). Together, these studies hinted that LDL has deleterious results in colorectal cancer development.

A case study in Japan showed a significant positive association between elevated levels of plasma ox-LDL and risk of colorectal cancer (102). ox-LDL and oxidative stress may increase the risk of obesity-related colorectal cancer *via* NF-κB signaling and could be used as potential predictive and prognostic biomarkers for obesity complicated with colorectal cancer (103). Other research has found LOX-1 to be more directly linked to the risk of colorectal cancer compared to ox-LDL (104). One study on the involvement of LOX-1 in colorectal cancer has shown that LOX-1 expression is increased in 72% of human colon carcinomas, and overexpressed in 90% of highly aggressive and metastatic tumors (104). Furthermore, LOX-1 expression is positively correlated with cancer stage and grade than healthy counterparts (105). It is worth noting that a recent study reported that the serum sample and 100 tissue samples from 238 colorectal cancer patients showed high levels of LOX-1 compared with those who present low serum levels, high levels of LOX-1 determine a poorer OS and prognosis of patients. Therefore, LOX-1 may help in liquid biopsy detection and cancer diagnosis and treatment under the premise that cancerous tissues are not available (106). LOX-1 takes its effects in colorectal cancer *via* upregulating of VEGF‐A165, HIF-1α, and β‐catenin, which are involved in cell migration and metastasis (107).

### LDL and ox-LDL in Pancreatic Cancer

pancreatic cancer is one of the most devastating malignancies, with a 5-year OS rate of less than 5% (108). Currently, treatment is dependent on surgical resection. However, only about 25% of pancreatic cancer patients are eligible for surgical resection due to pancreatic cancer invasion (109). Genome-wide association studies (GWAS) show that genetic factors are the primary risk factors related to pancreatic cancer; moreover, smoking, diabetes, drinking, obesity, chronic pancreatitis, and diet are all known risk factors for pancreatic cancer (110–112). A study found that genetically higher levels of LDL-C were associated with pancreatic cancer (113). As study showed that lipoprotein metabolic processes, in particular cholesterol uptake, are activated in the tumor. These metabolic processes increase the amount of cholesterol and the expression of LDLR in pancreatic tumor cells. Clinical data suggest that overexpressed LDLR is related to a high recurrence of pancreatic cancer (114, 115). High cholesterol intake is associated with an increased risk of pancreatic cancer (116), and knockdown of LDLR in patients’ cells greatly reduces cholesterol uptake and alters its distribution, decreases cancer cell proliferation, and limits the activation of the ERK1/2 survival pathway. LDL-C can promote the proliferation of pancreatic cancer cells by activating the STAT3 pathway and upregulating the levels of oncogenes such as *Bcl-2, Bcl-xL, survivin* controlled by this transcription factor in pancreatic cancer cells (49). These findings suggest that LDLR can be a novel metabolic target in limiting patients progression (114, 115).

LOX-1 is overexpressed in pancreatic cancer tumors compared with adjacent normal tissues, stimulates the migration of pancreatic cancer cells and invasion of lymph nodes by inducing EMT, and has been associated with higher tumor node metastases (TNM) staging and poorer OS (117). Recent study has found that ox-LDL transforms into a glycolytic phenotype by promoting metabolism and inducing cytoprotective autophagy, thereby making pancreatic cancer cells resilient or resistant (118) LOX-1 can up-regulate the expression of c-Myc, and the transcription of high mobility group AT-hook 2 (HMGA2). HMGA2 is up-regulated in many cancers, which can regulate cell proliferation and differentiation, as well as promote metastasis (119). Furthermore, LOX-1 is associated with pancreatic cancer drug resistance, the long-chain non-coding RNA GSTM3TV2 can up-regulate LOX-1 and promote the resistance of pancreatic cancer cells to gemcitabine (120). These findings suggest the potential role of ox-LDL receptors in the development of pancreatic cancer.

### LDL and ox-LDL in Prostate Cancer

Prostate cancer is the second most common malignancy worldwide in men (121). The growth of prostate cancer cells depends on the steroidal hormone androgen. Cholesterol is a common precursor of steroid hormones and plays a vital role in prostate differentiation and growth (120, 122). Epidemiological studies have shown strong or weak correlations between differences in plasma LDL-C concentration and the incidence of prostate cancer, though some are contradictory. Several studies failed to find any association between LDL-C and aggressive prostate cancer risk (123, 124), while a sizeable Mendelian randomization study showed a weak association between higher LDL-C levels and an increased risk of aggressive prostate cancer (125). In contrast, in another prospective study from the Netherlands, there is a positive association between prostate cancer risk and serum concentrations of LDL-C. Moreover, LDL-C is associated with higher cancer prevalence and more advanced tumor phenotypes (122, 126–128) These divergent results may be explained by the heterogeneity in the approaches used and significant differences in the follow-up of these studies.

Incubation of prostate cancer cells with LDL can significantly increase its proliferation, migration, and invasion (129). In addition, LDL-mediated effects on proliferation of prostate cancer cells are caused by PTEN loss and activation of Akt and ERK signaling pathways, and further activates SREBP, upregulating LDLR leading to cholesterol accumulation and cholesterol ester production (130). Moreover, LDL-C has been associated with higher cancer prevalence and more advanced tumor presentation. In the TRAMP mouse model, an autochthonous model of prostate cancer, hypercholesterolemia is shown to result in increased tumor volume and progression as well as increased tumor incidence and metastases to the lung (131). Furthermore, increased activation of *de novo* synthesis of cholesterol in tumor epithelial cells and influx of LDL from the surroundings tissues *via* LDL-R and SR-B1 promoted the bone metastasis of prostate cancer (132). It is noteworthy that normal cholesterol feedback of LDLR messages and protein is lost in prostate cancer (44). Because of the lack of LDLR feedback regulation, prostate cells obtain more essential fatty acids and increase prostaglandin 2 synthesis, leading to uncontrolled growth of prostate cancer cells (133). These studies suggest that prostate cancer may rely on cholesterol for metabolism and that low levels of LDL in cancer reflect the highly invasive nature of tumors. This suggests that LDLR may be an attractive therapeutic target for prostate cancer cells (134).

Elevated plasma ox-LDL levels and LOX-1 expression may indicate advanced prostate cancer and lymph node metastasis. The cell signaling pathway in human prostate cancer cells (PC-3) treated with ox-LDL analyzed by phosphorylated protein chip is of importance. Ox-LDL can affect a variety of signaling pathways of PC-3 cells, including β-catenin, cMyc, NF-κB, STAT1, STAT3, and apoptosis-related signaling pathways (including P27 and caspase-3), which affects the proliferation, migration, and invasion of prostate cancer cells, and *in vitro* experiments confirmed this (135). ox-LDL contributes to tumor progression through LOX-1 activation. Ox-LDL significantly triggers LOX-1 significantly and proportionally increases the expression of pro-angiogenic markers VEGF, MMP-2, and MMP-9, thereby promoting tumor metastasis (136). In addition, LOX-1 activated by ox-LDL reduces the expression of epithelial markers (*E-cadherin* and *plakoglobin*) and the expression of mesenchymal markers (v*imentin, N-cadherin, snail, slug*, etc), which lead to EMT that can further induce the invasion and migration of prostate cancer cells (20). All these observations suggest the use of ox-LDL and LOX-1 as a therapeutic target for prostate cancer.

### LDL and ox-LDL in Renal Cancer

Renal cancer is a common type of human malignancies. Clear cell renal carcinoma (ccRCC) is the primary subtype of renal cancer, characterized by abnormal lipid accumulation of cholesterol, cholesterol esters and triglycerides (137). Although abnormal LDL level is related to increased cancer risk (138), the correlation between high LDL levels and renal cancer risk is not consistently observed in clinical studies. Several recent studies have focused on the relationship between LDL and renal cancer and have come to conflicting conclusions. Two studies reported that LDL is elevated in renal cancer and is positively associated with cancer risk (138, 139). While contrary to the results of the study conducted by Zhang et al. this could be explained by the fact that the data in control groups differed between the two studies, and the serum lipid levels in the Zhang et al. controls were much higher. Different dietary patterns and lifestyles in north and south China may account for the discrepancy (140). It has been proved by *in vitro* and *in vivo* experiments that LDL-C decreases the anti-tumor effect of tyrosine kinase inhibitor (TKI) on renal cancer and endothelial cells by activating the PI3K/Akt pathway (50). Surprisingly, low LDLR expression was found in renal cancer subtypes of ccRCC cells, while SR-BI expression was significantly increased. High cholesterol levels in ccRCC were partly related to SR-BI-mediated HDL uptake (141, 142). The results of large-scale clinical trials indicated a direct correlation between LDL-C and renal cancer risk, encouraging more basic research in the future to elucidate the potential mechanisms of those correlations.

There is not yet an epidemiological study examining ox-LDL and renal cancer, while *in vitro* analysis of ccRCC showed that LOX-1 is expressed both in the cytoplasm and in the nucleus (143). It is known that diet-induced hypercholesterolemia increases the expression of LOX-1 in renal arterioles, and subsequently facilitates the uptake and cytotoxicity effects of ox-LDL (144) Ox-LDL enhanced the LOX-1 expression in tubular epithelial cells in a dose-dependent manner within a certain concentration range and mediates EMT progression in rat renal tubular epithelial cells NRK-52E. Moreover, HK-2 cells derived from normal kidneys exposed to ROS at a non-cytotoxic level for a long term showed increased proliferation, anchored independent growth, and enhanced tumorigenicity in nude mice (145). These studies provide direct evidence for the malignant transformation of renal tubular epithelial cells induced by oxidative stress.

### LDL and ox-LDL in HCC

HCC is the most prevalent cancer with a poor prognosis worldwide (146). Lipid seems to play a fundamental role in the development and progression of HCCs (147). As a key organ in lipid metabolism, liver is involved in the production of apolipoproteins, endogenous lipids and lipoproteins, which depend on the integrity of biofunctions of liver. Therefore, liver function in patients with HCC is significantly impaired, resulting in a distinctly abnormal patterns of serum lipids and lipoproteins (148). Multiple studies have shown that a decrease in both plasma HDL and LDL was slightly to significantly in HCC patients (149, 150). A large nationwide population-based study in South Korea showed that low lipid profile is an independent risk factor and preclinical marker of HCC (151). Similarly, in a report in Japan, low LDL was associated with increased mortality of HCC (152). However, there are some reports that the changes in lipoprotein levels and their prognostic significance in HCC are contradictory, and elevated LDL predicted a poorer prognosis for patients with HCC (153). Another study showed that low plasma HDL, high plasma LDL, and especially the combination of two, were significantly related to more aggressive HCC phenotype and the combination was significantly associated with a higher hazard ratio for death (154). There seems to be a two-way process because the presence of HCC is related to the aforementioned changes in plasma lipid. Low lipid profiles may reflect the degree of liver damage. When HCC occurs, the metabolism and synthesis of cholesterol are impaired, resulting in a decrease in plasma cholesterol levels (155). Meanwhile, the cancer cells increase cholesterol consumption to maintain faster proliferation, and changes in lipid content can alter HCC biological functions (154).

Previous study showed that serum cholesterol might promote the expression of *VEGF*, *MMP-2*, and *MMP-9* by activating the NF-κB signaling pathway in HCC cells, indicating the pro-inflammatory effects of cholesterol (156). In non-alcoholic fatty liver disease mice, a high-fat diet can induce HCC. Following initiation of the obesogenic diet, the mice developed obesity, insulin resistance, hypertriglyceridemia, and elevated LDL-C, and eventually developed HCC (157, 158). Besides, LDLR and cholesteryl ester levels are higher in the murine HCC tissues (159). Similarity, as described in human HCCs, LDLR is also up-regulated in cancer cells and stimulates cell proliferation (160).

Activation of oxidative stress is another key pathogenic mechanism. Plasma malondialdehyde and ox-LDL levels are significantly increased in HCC patients, and the oxidative stress was usually reversed after HCC resection (161). In addition, the uptake of ox-LDL *via* the CD36-Nogo-B-YAP pathway consequently drives the development of NAFLD-associated HCC (162).

### LDL and ox-LDL in Ovarian Cancer

Ovarian cancer is the most lethal gynecological malignancy in women. Patients with early ovarian cancer usually do not have cancer-specific symptoms, as a result, most are diagnosed with advanced ovarian cancer (163). Epidemiological studies of ovarian cancer risk and lipid levels are contradictory. A recent meta-analysis showed that the differences in plasma LDL-C between ovarian and non-ovarian cancer patients are not significant (164). In the Mendelian randomization analysis of 22,406 patients with invasive epithelial ovarian cancer, no association was found between the genetic variation that controls circulating LDL-C and the risk of epithelial ovarian cancer (165). While in a retrospective clinical study involving 1,550 ovarian cancer patients that assessed blood lipid characteristics, it was found that compared to the benign ovarian tumor group, levels of LDL-C and TC in the ovarian cancer group were significantly lowered (166). It should be noted that intracellular cholesterol levels were found to be elevated in high-grade serous ovarian cancer cells and malignant ascites (167). This might be explained by the highly malignant nature of ovarian cancer, which progresses quickly. Rapid tumor growth requires large amount of consumption of cholesterol and which subsequently leads to decreased plasma levels of LDL-C (168). Furthermore, ovarian cancer patients with a low blood cholesterol level at the time of diagnosis show improvement in blood cholesterol level after successful primary surgery and chemotherapy (169).

Clinical case-control studies suggest that plasma ox-LDL levels are associated with an increased breast and ovarian cancer risk. Ox-LDL is mitogenic to ovarian cancer cells. ox-LDL at a dose of 0.1 μg/mL stimulates the proliferation of ovarian cancer cell lines CAOV3 and SKOV3 and reduces the sensitivity of cancer cells to cisplatin (85, 86). Alternatively, LXR agonists and fluvastatin can reverse the effect of ox-LDL, suggesting LXR ligands and statins may be effective in the treatment of ovarian cancer (60). While the data remain limited, available evidence suggests that abnormal lipoprotein profiles might promote ovarian cancer development.

### LDL and ox-LDL in Gastric Cancer

Gastric cancer is the second most common cancer worldwide and the third leading cause of cancer-related deaths (121). A prospective study reveals that higher LDL-C to be a risk factor for gastric cancer, and compared with healthy controls, serum LDL-C levels were lower in patients with gastric cancer; whereas, LDL-C levels in gastric cancer tissues are higher than normal tissues (170). More importantly, increased LDL levels favor cancer metastasis to lymph nodes (171). It is possible that diagnosed cancer at its late stage may cause a reduction in serum LDL-C levels due to a rapid cell division. Alternatively, through the LDL-lowering effect, recent studies have demonstrated that statins reduce the risk of gastric cancer by inhibiting cancer cell growth and cell death (172). Despite multiple researches supporting the prognostic value of serum lipid levels in gastric cancer, a solid association has not been confirmed (170, 173).

Recent studies showed that the blood levels of ox-LDL increased in gastric cancer patients, and LOX-1 was up-regulated in gastric cancer tissues (21, 174). High expression of LOX-1 is not only related to cancer invasion and lymph node metastasis but is also associated with TNM stage and OS reduction (174). *In vivo* and *in vitro* experiments demonstrated that ox-LDL could activate the NF-κB signaling pathway *via* LOX-1, with subsequent upregulation of VEGF-C and promotion of the lymphatic metastasis of gastric cancer (21). Besides, LOX-1 promotes cell migration and invasion by activating the PI3K/Akt/GSK3β pathway and then enhances the EMT process of gastric cancer cells (174). These results suggest that LOX-1 may represent a promising prognosis factor for gastric cancer and serves as a novel molecular target for gastric cancer therapies.

LDL and ox-LDL levels and their possible effects on the development of those types of cancers are summarized in **Tables 1**, **2** below, and different roles of LDL and ox-LDL in cancer progression summarized in **Table 3**.

**Table 1:** LDL levels and their possible effects on the development of selected types of cancer.

Cancers	Study design	Experimental subjects	LDL level	Main effects	Ref
Breast cancer	Mendelian randomization	>400,000 cases	↑	Increase cancer risk	(75)
	Prospective	244 cases	↑	Promote tumor growth and differentiation	(74)
	*In vitro*	HTB20, 4 T1, HTB126, MDA MB 231, MCF7, HS578T, MDA MB 468 cell lines		Promote proliferation, migration, invasion, angiogenesis	(48, 83)
	*In vivo*	Female BALB SCID, NOD SCID and BALB/C mice; (Rag1^−/−^/LDLR^−/−^ and Rag1^−/−^/ApoE^−/−^ mice	↑	Promote tumor growth	(84)
Colorectal cancer	Case-cohort	34148 cases	↑	Increase cancer risk	(96)
	Retrospective	453 cases	↑	Promote distant metastasis	(98)
	*In vitro*	SW480, LoVo and RKO cell lines		Promote migration	(55)
	*In vivo*	AOM/DSS-treated mice	↑	Enhance intestinal inflammation	(55)
Pancreatic cancer	Mendelian randomization	8769 cases	↑	Increase cancer risk	(175)
	*In vitro*	PK4A cell line		Promote proliferation	(115)
	*In vivo*	Male Pdx1-Cre, Ink4a/Arf*^fl/fl^* and LSL-Kras*^G12D^* mice	↑	Increase recurrence risk and drug resistance	(115)
Prostate cancer	Cohort	2842 cases	↑	Increase cancer risk	(176)
	Cross-sectional	190 cases	↑	Increase cancer risk	(127)
	*In vitro*	LNCaP and VCaP cell lines		Promote proliferation and migration	(129)
	*In vivo*	C57Bl/6J mice	↑	Promote tumor growth	(131)
Renal cancer	Retrospective	362 cases	↑	Increase cancer risk	(139)
	Case-control	Cancer patients:550 cases Control:570 cases	↑	Increase cancer risk	(138)
	*In vitro*	SK-45 and PNX0010 cell lines		Resist chemotherapy	(50)
	*In vivo*	Male C.B17/Icr-scid mice	↑	Promote tumor growth	(50)
Hepatocellular carcinoma	Prospective	26891 cases	↓	Increase cancer risk	(151)
	Cohort	16217 cases	↓	Increase mortality	(152)
	*In vitro*	HepG2 and Huh7 cell lines		Promote inflammation	(156)
	*In vitro*	C57BL/6J mice	↑	Increase cancer risk	(177)
Ovarian cancer	Retrospective	267 cases	↑	Improve 5-year RFS	(168)
	Case-control	Cancer patients: 22406 cases Controls:40941 cases		No significant associations	(165)
Gastric cancer	Case-control	Cancer patients: 412 cases Controls: 2934 cases	↑	Increase cancer risk	(170)
	Cross-sectional	205 cases	↑	Predict metastasis risk	(171)

↑: up-regulation, ↓: down-regulation.

**Table 2:** LDL levels and their possible effects on the development of selected types of cancer.

Cancers	Study design	Experimental subjects	ox-LDL level	Main effects	Ref	
Breast cancer	Case-control	Cancer patients:32 cases Controls: 30 cases	↑	Increase cancer risk	(86)	
	*In vitro*	MCF10A cell line		Promote tumorigenesis	(88)	
Colorectal cancer	Case-control	Cancer patients: 161 cases Controls: 395 cases	↑	Increase cancer risk	(102)	
	Retrospective	52 cases	↑	Increase cancer risk	(103)	
Pancreatic cancer	*In vitro*	KLM-1 cell line		Promote tumorigenesis and proliferation	(118)	
Prostate cancer	Retrospective	75 cases	↑	Promote Gleason score and lymph node metastasis	(135)	
	*In vitro*	LNCaP, PC-3, C4-2, C4-2B and DU-145		Promote proliferation, migration, and invasion	(20, 135, 136)	
	*In vivo*	Male BALB/c mice	↑	Enhance tumor angiogenesis	(136)	
Hepatocellular carcinoma	Cross-sectional	50 cases	↑	Induce carcinogenesis	(161)	
	*In vivo*	Female athymic nude mice and C57BL/6 mice		Promote tumorigenesis	(162)	
Ovarian cancer	Case-control	Cancer patients:32 cases Controls:30 cases	↑	Predict cancer risk	(86)	
	*In vitro*	CAOV3, SKOV3 cell lines		Promote proliferation and drug resistance	(86)	
Gastric cancer	Retrospective	17 cases	↑	Promote lymph node metastasis	(21)	
	*In vitro*	HGC-27 and MGC-803 cell lines		Promote metastasis	(21)	
	*In vivo*	Female BALB/c nude mice	↑	Promote lymph node metastasis	(21)	

↑: up-regulation, ↓down-regulation.

**Table 3:** Summary of the role of LDL and ox- LDL in cancer progression.

Lipoproteins	Receptors	Mechanism	Effects on cancer	Ref
LDL	LDLR	Inhibit PD1/L1 and γδ T cells	Anti-tumor therapy resistance	(51, 53)
		Up-regulate Stemness genes	Enhance cell stemness	(55)
		Activate Akt/ERK2, p38/MAPK, PI3K/Akt/mTOR signaling pathways; decrease adhesion molecules expression	Proliferation and metastasis	(48, 50, 55, 82)
		Activate STAT3 signaling	Invasion	(49)
ox-LDL	LOX-1, CD36	Induce DNA damage by ROS, activate miR-210 expression	Inflammation and mutagenesis	(39, 57, 58)
		Activate POX to up-regulated beclin-1 activate miR-155	Autophagy	(63, 64)
		Activate NF-κB target genes *VEGF, MMP-2, MMP-9* by binding with LOX-1; up-regulate cytokines CT-1 by accumulating ox-LDL oxysterol metabolite	Proliferation, invasion, and angiogenesis	(39, 60)
		Activate PI3K/Akt/GSK3β signaling pathway	EMT and migration	(174)

## Targeting LDL and ox-LDL for Cancer Therapy

Given the association between high cholesterol levels and cancer progression, treatments aiming to lower serum cholesterol levels may have beneficial effects on cancer. Considering the role of LDL/ox-LDL in cancer occurrence, progression, and metastasis, targeting the receptors of LDL and ox-LDL may be a clinically valuable therapeutic strategy. It has been shown that the inhibition of LDLR activity in pancreatic and breast cancer cells can significantly reduce cholesterol absorption and subsequent inhibition of cell proliferation (84, 115). Many malignant cancers have an increased demand for lipoprotein due to the requirement for lipids for the rapid proliferation of the tumors, which is met by the increased availability of LDL through upregulation of LDLR. LDLR is not downregulated, especially in prostatic cancer with the elevation of LDL level in the body (44). Thereby, taking LDL as a carrier, anticancer drugs can target tumor cells more precisely and effectively. Other studies have shown that targeting ox-LDL-related receptors has the potential to reduce metastasis in a variety of *in situ* cancer models. In xenograft models, the inhibitory effect of LOX-1 significantly inhibits the formation of metastasis in tumor-bearing mice (107). Thus, LDL and ox-LDL and their receptors play an essential role in the process of cancer treatment and can be used as an effective adjunct to current cancer treatment.

Traditional cholesterol-lowering drugs such as statins competitively inhibit the endogenous cholesterol synthesis by targeting the rate-limiting enzyme HMG-CoA reductase and block hydroxymethyl, to control the biosynthesis of cholesterol in the cell, which can effectively reduce LDL-C (178–180). In recent years, statins have been considered anti-cancer drugs (181). Statins are associated with significantly reduced risk of breast cancer, colorectal cancer, ovarian cancer, pancreatic cancer, lung cancer, and lymphoma, Statins have been shown to inhibit tumor growth at clinically relevant doses and diminish metastasis in animal models (182, 183). Besides, statins reduce mortality and the risk of prostate-specific antigen (PSA) recurrence in a dose-dependent manner after prostatectomy (184), as well as improve OS in patients with metastatic renal cancer (25.6 months vs. 18.9 months) (185).

Mevalonic acid, a the precursor of non-steroidal isoprenoids, is a lipid attachment molecule for small G proteins, such as Ras, Rho, and Rac, and has been implicated in various aspects of tumor development and progression (179). Statins have been shown to inhibit the proliferation of breast cancer cells by suppressing FPP and GGPP modifications and activation of Ras, Rac, and Rho, small GTPases. In addition, statins inhibit cancer cell growth by inducing apoptosis through activation of Bax and down-regulation of the levels of anti-apoptotic protein Bcl-2 (186). However, the effects of statins on angiogenesis are divergent. Statins induce angiogenesis at low doses, while an opposite effect is observed at higher doses (187). Furthermore, statins can inhibit the viability and proliferation of cancer cells by blocking various signaling pathways, such as PI3K/ATK and MAPK, and may improve the efficiency of chemotherapy when used in combination with other chemotherapeutic agents (184). However, statins with different solubility show different effects on cancer therapy. Lipophilic statins have better anticancer activities than hydrophilic statins; this may be partly attributed to their better ability in diffusing into extrahepatic tumors. In this regard, it is proposed that hydrophilic statins are not effective in inhibiting extrahepatic HMG-CoA reductase, and are thus ineffective in reducing cancer susceptibility (188).

## Conclusion and Prospects

Most cancers show a high demand for cholesterol, LDL or ox-LDL to maintain rapid growth and survival. LDL and ox-LDL play divergent roles in different types of cancers. In certain types of cancer at their early stages, elevated plasma LDL-C levels in patients are observed, such as in colorectal cancer patients. However, plasma LDL-C was reported to be lowered in patients with metastatic cancer. The relationship between low plasma LDL-C levels and cancer can be explained by increased uptake of cholesterol from plasma by malignant cells to meet their own proliferation needs. Elevated plasma levels of either LDL or ox-LDL are positively correlated with the progression of breast cancer, colorectal cancer, and pancreatic cancer, but no such correlation has been found between ox-LDL and thyroid or nasopharyngeal cancers, which may be due to the differing needs for cholesterol in different cancer types. LDLR and LOX-1, as receptors for LDL and ox-LDL, respectively, are overexpressed in a variety of cancers and are associated with accelerated cancer progression. However, there is currently limited data on whether high expression levels of these receptors will be present increase the risk of cancer. The connection seems clear, and it is necessary to determine the correlation between LDL and ox-LDL-related receptors and cancer risk. Statins have multiple anti-cancer effects such as inhibition of cancer cell proliferation, promotion of cancer cell apoptosis, and enhancement of the efficacy of chemotherapy drugs, and liposoluble statins may be more suitable for cancer treatment.

Epidemiological studies have shown that cancer is often accompanied with metabolic diseases, such as hypertension, hyperlipidemia, and diabetes. Cancer patients often have high-risk habits such as smoking, drinking, and a high-fat diet. Such confounders may lead to elevated LDL/ox-LDL levels in cancer patients. Identifying the causes of elevated LDL and/or ox-LDL levels may help to elucidate novel therapies for reducing LDL and/or ox-LDL levels in cancer patients. Complex feedback loops regulate cholesterol homeostasis, by only inhibiting one pathway of cholesterol metabolism might have little effect on tumor growth. With the increased discovery of inhibitors targeting cholesterol metabolism, the effects of combination therapy simultaneously block cholesterol synthesis, uptake, esterification, or cancer trafficking should be further explored. Despite exciting progress in this field, many fundamental questions remain to be addressed, such as could some drugs currently used for the treatment of metabolic diseases be repurposed as anti-tumor drugs? What is the most effective combination way to treat a particular type of cancer cells with different approaches? These unresolved issues reflect the urgent need for more research on the mechanism of cholesterol metabolism in cancer.

Taken together, these studies strongly suggest that LDL, ox-LDL, as well as their receptors, play important roles in tumorigenesis and cancer development. Lowering LDL and ox-LDL levels may be a novel therapeutic strategy to prevent cancer progression.

## Author Contributions

All authors contributed to the development of this review article. Critical analysis and review of the literature were performed by C-FD. The manuscript was written by C-FD and NZ with revisions provided by T-JZ, H-FL, JG, D-FL, LQ.

## Funding

This work was supported by the National Natural Sciences Foundation of China (81973668, 81774130); the National Science Fund of Hunan Province for Distinguished Young Scholars (2018JJ1018); the Key Project of the Educational Department of Hunan Province (20A375); the Scientific Research Project of Changsha Science and Technology Bureau (No. kq2004060) and the First-Class Discipline of Pharmaceutical Science of Hunan.

## Conflict of Interest

The authors declare that the research was conducted in the absence of any commercial or financial relationships that could be construed as a potential conflict of interest.

## Publisher’s Note

All claims expressed in this article are solely those of the authors and do not necessarily represent those of their affiliated organizations, or those of the publisher, the editors and the reviewers. Any product that may be evaluated in this article, or claim that may be made by its manufacturer, is not guaranteed or endorsed by the publisher.

## Abbreviations

ABCA1, ATP binding cassette transporters A1; ABCG1, ATP binding cassette transporters G1; ACAT-1, coenzyme a-cholesterol acyltransferase-1; BMI, Body mass index; ccRCC, clear cell renal carcinoma; CD36, cluster of differentiation 36; CDHR, cadherin-related family member3; CT-1, carditorphin 1; EMT, Epithelial mesenchymal transformation; ER, estrogen receptor; FAK1, focal adhesion kinase 1; FPP, farnesyl pyrophosphate; GGPP, geranylgeranyl pyrophosphate; GWAS, genome-wide association studies; HCC, hepatocellular carcinoma; HER2, epidermal growth factor receptor 2; HIF-1α, hypoxia-Inducible factor 1-Alpha; HMGA2, high mobility group AT-hook 2; HMGCR, 3-hydroxy-3-methylglutaryl coenzyme A reductase; LDL, low-density lipoprotein; LDL-C, LDL-cholesterol; LDLR, LDL receptor; LOX-1, lectin-like oxidized low-density lipoprotein receptor-1; LXR, liver X Receptor; miR-155, microRNA155; miR-210, microRNA-210; MMPs, matrix metalloproteinases; NF-κB, nuclear factor kappa-B; OS, overall survival; ox-LDL, oxidized low-density lipoprotein; PCSK9, proprotein convertase subtilisin/kexin type 9; POX, proline oxidase; PR, progesterone receptor; PSA, prostate-specific antigen; RAC1, Ras-related C3 botulinum toxin substrate 1; RNS, reactive nitrogen species; ROS, radical oxygen species; SPRED2, sprout-related EVH1 domain 2; SQLE, squalene epoxidase; SREBP, sterol regulatory element-binding proteins; SRs, scavenger receptors; TKI, tyrosine kinase inhibitor; TNM, tumor node metastases; VEGF, vascular endothelial growth factor.
